# Comparative Value of Simple Inflammatory Markers in the Prediction of Left Ventricular Systolic Dysfunction in Postacute Coronary Syndrome Patients

**DOI:** 10.1155/2009/826297

**Published:** 2009-05-25

**Authors:** Panagiotis Aggelopoulos, Christina Chrysohoou, Christos Pitsavos, Lambros Papadimitriou, Catherine Liontou, Demosthenes Panagiotakos, Eleftherios Tsiamis, Christodoulos Stefanadis

**Affiliations:** ^1^First Cardiology Clinic, School of Medicine, University of Athens, 115 28 Athens, Greece; ^2^Department of Nutrition Science and Dietetics, Harokopio University, 176 71 Athens, Greece

## Abstract

*Objectives*. We sought to assess the comparative value of inflammatory markers on the occurrence of left ventricular systolic dysfunction (LVSD) after an acute coronary syndrome (ACS). *Methods*. During 2006–2008, 760 patients with an ACS were enrolled. C-reactive protein (CRP) and white blood cell (WBC) count were measured during the first 12 hours of hospital admission. *Results*. CRP levels and WBC count were significantly higher in those who developed LVSD compared to those who did not. The analysis revealed that a 10 mg/dL increase of CRP levels and a 1000/*μ*L increase in WBC are associated with a 6% and a 7% increase in the likelihood of developing LVSD, respectively. Furthermore, WBC count at entry and CRP have almost the same predictive value for development of LVSD after an ACS (*R* ^2^ = 0.109 versus *R* ^2^ = 0.093). *Conclusions*. Serum CRP levels and WBC count at entry are almost equally powerful independent predictors of LVSD, after an ACS.

## 1. Introduction

Heart failure could be described as a modern plague, as it is the leading cause of hospital admissions in the United States [1]. Among other diseases leading to heart failure, coronary heart disease is known to be the main cause, often implicated by acute heart failure [2]. The relationship between inflammation and heart failure has been demonstrated in several studies. Among other inflammatory markers, leukocyte count for men and women hospitalized for any type of acute coronary syndrome has been recognized as an independent predictor of hospital death and development of heart failure [3]. Furthermore, inflammatory markers such as CRP, IL-6, and TNFa, are associated with increased risk of congestive heart failure in older people without prior myocardial infarction [4, 5]; while CRP is strongly and independently associated with the occurrence of new onset heart failure [6–11]. 

The assessment of the risk for developing left ventricular systolic dysfunction and eventually systolic heart failure in ACS patients is of considerable clinical and public health importance; while the comparative role of CRP and WBC count in occurence of left ventricular systolic dysfunction in the setting of ACS is still unclear. 

The aim of this study is to investigate the implication of inflammation, characteristically expressed by the levels of CRP, and white blood cell count, on the occurrence of LVSD in the acute coronary syndrome (ACS) process, thus providing insights in the contribution of inflammation to the deterioration of the systolic function of the heart in the setting of ACS. Moreover, we aim to estimate the comparative predictive value of the two markers assessed.

## 2. Methods

### 2.1. Population of the Study

During 2006–2008, we enrolled 798 consecutive patients who developed an ACS event. From the above we finally included in the study 283 males (64 ± 14 years) and 73 females (71 ± 12 years) postacute coronary syndrome patients with impaired ejection fraction, defined as below 40% and 317 males (62 ± 11 years) and 87 females (67 ± 11 years) with normal ejection fraction, defined as above 50%. The total number of patients were therefore 760, while the rest 38 exhibited an ejection fraction of 40–50% and were not included in the following analysis. Patients that were estimated as having an EF of 40–50% were not included, as this is only mild impairment of ventricular function. The echocardiographic evaluation of the left ventricular function that was used in this analysis was the one performed at the discharge day, in order to overcome the stunning of the first days after the acute coronary event.

### 2.2. Diagnosis of ACS

At entry a 12-lead electrocardiogram was performed, and clinical symptoms were evaluated in all patients, by a cardiologist of the study. Based on the electrocardiographic findings patients were classified as having ST-segment elevations, non-ST segment elevations, or other electrocardiographic abnormalities. Moreover, troponin I measurements were performed to detect evidence of myocardial cell death. We included only cases with diagnosis of ACS (acute myocardial infarction (MI) or unstable angina (UA)). In particular, acute myocardial infarction was defined according to the latest guidelines [12]; while unstable angina was defined by the occurrence of one or more angina episodes, at rest, within the preceding 48 hours, corresponding to class III of the Braunwald classification [13].

Using a Hewlett Packard 5500 Sonos with a multifrequency transducer (2, 5-4 MHz) we evaluated the systolic function of the left ventricle during the first 48 hours and at the discharge day. Systolic heart failure was defined as left ventricular ejection fraction at the discharge day below 40%, according to the recent European Society of Cardiology guidelines for the diagnosis and treatment of acute heart failure [1]. For this reason we excluded from this study those patients with an ejection fraction of the left ventricle above 40% and below the normal limits of 50%, as those represent mild impairment of left ventricular function at the discharge day.

The study was approved by the Medical Research Ethics Committee of our Institution and was carried out in accordance with the Declaration of Helsinki (1989) of the World Medical Association, while each patient gave written consent for participating.

### 2.3. Other Clinical and Biochemical Characteristics

In all patients a detailed medical history was recorded, including previous hospitalization for cardiovascular disease (i.e., coronary heart disease, stroke, or other cardiovascular disease), presence and management of hypertension, hypercholesterolemia, renal failure, and diabetes mellitus. Moreover, we recorded patients' medical family history. In addition to troponin I, we also measured the MB fraction of creatine phosphokinase (CPK), urea, and uric acid. The biochemical evaluation was carried out in the same laboratory that followed the criteria of the World Health Organization Reference Laboratories. Total-, high-density lipoprotein, cholesterols, blood glucose, and triglycerides were also measured in all participants, using colorimetric enzymic method in a Technicon automatic analyzer RA-1000 (*Dade-Behring Marburg GmbH, Marburg, Germany*). Low-density lipoprotein cholesterol was calculated using the Friedewald formula: total cholesterol—HDL cholesterol—1/5 × (triglycerides). An internal quality control was in place for assessing the validity of cholesterol and triglycerides methods. The intra- and interassay coefficients of variation of cholesterol and triglycerides levels did not exceed 4%.

 White blood cell count was measured at the initial blood test at entry, and C-reactive protein levels were measured during the first 12 hours of hospitalization, specifically at 9:00 a.m. of the next morning. CRP levels were measured by nephelometry.

 Renal insufficiency was initially quantified by the baseline estimated creatinine clearance rate (CrCl). Based on baseline serum creatinine (Cr), the CrCl was calculated using the Cockcroft-Gault formula (14): CrCl = [[(140 − age) × weight] / (72 × serum creatinine)] for men, while for female gender, the result of the above equation was multiplied by 0.85 [14].

### 2.4. Demographic, Anthropometric, and Lifestyle Characteristics

Sociodemographic characteristics included age, sex, marital status, number of children, years of school, type of occupation. Height and weight were measured and to the nearest 0.5 cm and 100 g, respectively. Body mass index (BMI) was then calculated as weight (in kilograms) divided by height (in meters) squared. Physical activity was defined as any type of exercise (occupational or leisure) at least once per week during the past year. Other participants were defined as physically inactive. Current smokers were defined as those who smoked at least one cigarette per day or have stopped cigarette smoking during the past 12 months. Former smokers were defined as those who had stopped smoking more than one year previously. The rest of them were defined as never smokers or rare smokers.

### 2.5. Statistical Analysis

In this work continuous variables are presented as mean values ± standard deviation. The categorical variables are presented as absolute and relative (%) frequencies. Associations between continuous variables and groups of patients were evaluated through the analysis of variance (ANOVA), after controlling for equality of variances (homoscedacity). Associations between categorical variables were tested by the use of the chi-squared test, without the correction of continuity. Correlations between continuous variables were tested by the use of Pearson's correlation coefficient. Due to multiple comparisons we applied the Bonferroni correction to correct for the inflation of Type—I error. Logistic regression analysis was applied in order to evaluate the association between the investigated inflammatory markers and the development of LVSD, after various adjustments were made. Deviance residuals and Hosmer-Lemeshow criterion were used to test models' goodness-of-fit. All statistical calculations were performed on the SPSS version 12.0 software (SPSS Inc, Texas, Ill, USA).

## 3. Results

Table 1 illustrates the clinical characteristics of the patients according to the presentation or not of LVSD at discharge. Patients who developed systolic dysfunction had lower systolic and diastolic blood pressure at entry, higher entry levels of troponin I, glucose, uric acid, WBC count and serum CRP, lower creatinine clearance as well as higher prevalence of myocardial infarction as a discharge diagnosis. Furthermore, they had more extensive coronary artery disease according to their coronary angiogram, while there was no significant difference regarding smoking habits, physical activity status, hypertension, hypercholesterolemia, previous medication and previous history of CHD, body mass index, age, and sex. 

Then, we divided WBC count in tertiles (lower tertile < 8180/*μ*L and upper tertile > 11000/*μ*L) and we revealed that those patients in the upper tertile were younger, had more often myocardial infarction than unstable angina, had higher levels of troponin I at entry, had less often previous history of coronary heart disease, and were more often current smokers (Table 2). Similarly, we divided CRP levels in tertiles (lower tertile < 6.52 mg/L and upper tertile > 28 mg/L) and we revealed that those patients in the upper tertile were older, had more often myocardial infarction rather than unstable angina, had higher levels of troponin I at entry, and had lower levels of creatinine clearance (Table 3).

Logistic regression analysis, after controlling for sex, age, diagnosis of myocardial infarction, troponin I levels at entry, systolic and diastolic blood pressure levels at entry, and creatinine clearance, revealed that an increase per 10 mg/L of serum CRP levels independently increases by 6% the likelihood of developing LVSD after an acute coronary event (OR = 1.006^10^, *P* = .002) (Table 4). Moreover, after controlling for the same confounders as before, we revealed that an increase of 1000 count of WBC count at entry is independently associated with an increase by 7% of the likelihood of developing LVSD after an acute coronary event (OR = 1.00007^1000^, *P* < .001) (Table 4).

We used two logistic regression models using the same confounders each time and entering CRP the first time and WBC count afterwards. Comparing the *R* Square for the two logistic regression models, that is to say, the amount of variance explained by each model, we observe that WBC count seems to be slightly but not significant more powerful, in predicting the development of left ventricular systolic dysfunction, than CRP (*R* ^2^ = 0.109 versus *R* ^2^ = 0.093). (Table 4).

## 4. Discussion

In this study we revealed that higher values of white blood cell count are associated with higher probability of developing left ventricular systolic dysfunction after an acute coronary syndrome, independently of diagnosis of myocardial infarction or other common conditions that might contribute to this. We also demonstrated that CRP is an independent predictor of development of left ventricular systolic dysfunction after an ACS, as well, after controlling for common confounders (including myocardial infarction diagnosis). Although WBC count seems to be a slightly more powerful predictor for the development of systolic dysfunction than CRP, this difference was not statistically significant.

In the setting of acute coronary syndromes, inflammation is known to have a key role in disease progression and development of complications. We assessed left ventricular dysfunction occurrence using two widely used and easily measured markers: CRP is a proven marker of inflammation, yet the WBC count seem to hold its role in predicting poor prognosis in a manner that exceeds just inflammation.

### 4.1. WBC and Acute Systolic Heart Failure

It is well known that the leucocyte count is elevated in the setting of myocardial infarction. Leukocytes are the first cells drawn to an infarct area of necrosis trying to repair the tissue damage. As we revealed in our study that the initial WBC count seems to be higher in younger patients, who more frequently suffer from myocardial infarction rather than unstable angina, have higher heart rate at entry, have higher troponin I levels, have less frequently prior history of coronary heart disease, and are more often current smokers (Table 2). These correlations are more or less expected and form a patient profile that is previously linked to congestive heart failure occurence after an ACS [15], while smoking is recently also associated with WBC count and worse hospital and short-term prognosis in an analysis from GREECS study [16].

The leukocytes seem to mediate left ventricular systolic dysfunction in the setting of an acute coronary syndrome in many ways; elevated white blood cell count is associated with more extensive coronary artery disease, as assessed by coronary angiography [17]. Leukocytes are also associated with worse myocardial perfusion that can be due to microvascular plugging and abnormal leucocyte aggregation [18], a mechanism that might contribute to the “no-reflow’’ phenomenon. In another study of stable angina patients, neutrophil count was associated with coronary vascular stenosis complexity [19]. Another mechanism is that of myocardial infarct expansion that can be partly mediated through binding of the leucocytes through adhesion molecules to the microvascular endothelium. Moreover, leucocytes may mediate injury through releasing proteolytic enzymes and oxidative radicals. In experimental models, neutropenia was associated with smaller infarct sizes [20]. 

It appears that leykocytes may have an important role in tissue repair, but seem to enhance a portion of further tissue damage, thus explaining their contribution to the development of systolic dysfunction, regardless of the infarct itself.

### 4.2. CRP and Acute Systolic Heart Failure

It is previously shown that CRP is elevated in cases of myocardial infarction, as CRP rises in response to various inflammatory stimuli, including tissue necrosis and myocardial infarction [21, 22]. During destabilization of coronary heart disease, cytokines released by inflammatory cells mediate further inflammatory cell migration and cardiac repair and, particularly IL-6, mediates CRP elevation [23]. These correlations betwen CRP and myocardial necrosis were observed in our study as well (Table 3). Moreover, higher CRP levels were observed in older patients and in those with lower creatinine clearance.

CRP may be linked to left ventricular systolic dysfunction progression through three different mechanisms: its relation to the extent of coronary artery disease, its relation to thrombus burden, and the proinflammatory effect that may mediate further tissue damage in cases of myocardial infarction. CRP is reported to be associated to CAD extent in myocardial infarction patients [24] but nonetheless in unstable angina patients the correlation is reported to be weak [25]. It is best linked to the overall thrombus burden, as CRP is recognised to have procoagulant effects through stimulating tissue factor production. Furthermore, it is reported that CRP mediates further inflammatory stimulation and may be implicated in augmenting the infarct damage through the action of inflammatory cells [24].

 It is known that CRP concentration demonstrates diurnal variation that is statistically significant and may bias epidemiological studies [26, 27]. CRP levels in STEMI patients are higher during daytime, in contrast to nighttime. In our study, however, blood samples for CRP measurement were drawn at 9:00 a.m. for all patients, in both study groups, so diurnal variation is not expected to bias our results. As for seasonal heterogeneity, CRP concentration demonstrates only a tendency for higher values in the colder months that is moreover of borderline statistical significance [26]. Furthermore, CRP levels do not present significant fluctuations attributed to gender, phase of menstrual cycle, or use of a conventional needle for the drawing of blood samples [26]. In our study CRP measurements were performed within 1-2 hours of blood collection, so there is no significant delay in processing the sample [26].

Among the two inflammatory markers assessed in this work, the WBC count seems to have a slightly better predictive power for the development of left ventricular systolic dysfuncton after an ACS, when compared to CRP. CRP is related to systolic dysfunction after an ACS through three different mechanisms: that of total atherosclerotic burden which means that it is related to more extensive coronary vascular disease, that of thrombus burden which means that having procoagulant effects, may account for a portion of thrombus formation, and that of inflammatory escalation. On the other hand, leukocytes are implicated more actively and in more pathophysiologic steps than CRP; they are related to more extensive coronary vascular disease and they contribute to inflammatory escalation, oxidative stress, proteolytic enzymes release; microvascular plugging, lytic action of macrophages. This can partly explain the findings of another study that associates high neutrophil count (among all leucocyte subtypes) with higher mortality rates following an ACS, independently of CRP levels [9] and is partly explained by the various mechanisms of tissue damaging, apart from inflammation.

### 4.3. Study Limitations

In retrospective case-control studies, two main sources of systematic errors may exist, the selection and the recall bias. In order to eliminate selection bias we tried to set objective criteria, both for patients and controls having the same echocardiographic evaluation of left ventricular function obtained by two independent physicians at the discharge day, in order to eliminate the impact of stunning myocardium of the first days after the event. Also, in case-control studies, it is usually observed that patients who had a recent adverse event are more likely to place greater emphasis (overestimate) on several factors related to the disease than the control group (recall bias). In order to reduce this type of bias, we made an effort to obtain accurate information from the patients as well as from their relatives or their accompanying persons. Besides, the two groups were patients with different disease severity. Another limitation is that we have also included patients with previous history of coronary heart disease. These patients may have had impaired left ventricular function before the index event. In order to eliminate this bias we have included the information of previous known history of coronary heart disease in the multivariate analysis; the percentage of patients with previous CHD history had no significant difference between the two groups of patients.

The CRP assay was not a high sensitivity one, but this fact is not expected to influence our results, as all patients are suffering from an acute coronary syndrome and are, therefore, expected to exhibit elevated values of CRP.

Concerning the medical information, we tried to avoid recall bias by obtaining accurate and detailed data from subjects' medical records. However, over/under estimation may still exist, especially in the measurement of smoking habits and the onset of the investigated cardiovascular risk factors. Moreover, the coronary patients who died at entry or the day after were not included in the study. This bias could influence our results, but, since the proportion of deaths during the first two days was estimated between 2–4%, we believe that the inability to include the fatal events did not alter significantly our findings. Furthermore, regarding the potential effect of uncontrolled—unknown confounders, we tried to reduce it by using the same study base, both for patients and controls, but their influence on the outcome may still exist.

### 4.4. Conclusions

Elevated WBC and CRP levels are independent predictors of development of left ventricular systolic dysfunction after an ACS, regardless of several confounders including diagnosis of myocardial infarction. The WBC count seems to be slightly more predictive than CRP in this direction. Hopefully clinicians may consider the initial WBC count and CRP as almost equally powerful measures of possible left ventricular systolic dysfunction occurence, that may lead to heart failure. The inflammatory process may also be targeted for therapy, as this may provide some protection against heart failure occurrence.

## Figures and Tables

**Table 1:** Clinical characteristics of the patients according to systolic heart function.

	With LVSD	Without LVSD	*P*
*N*	356	404	
Age (yrs)	66 ± 14	63 ± 12	.17
Male sex (%)	80	78	.58
BMI (Kg/m^2^)	27.5 ± 4	28 ± 5	.14
Systolic blood pressure at entry	128.6 ± 29.2	133.6 ± 26	.013
Diastolic blood pressure at entry	75.5 ± 14	77.4 ± 13	.05
Heart rate at entry	81 ± 16	79 ± 16	.71
Diagnosis MI (%)	84	71	<.0001
Number of Vessel disease	2 ± 0.9	1.8 ± 0.9	<.0001
Previous CHD (%)	49	47	.62
Time delay (min)	548 ± 1953	651 ± 2702	.65
Troponin I at entry	40.28 ± 72.77	16.74 ± 39.43	<.0001
Uric acid (mg/dL)	6.69 ± 2.15	6.14 ± 2.02	<.0001
Blood glucose at entry (mg/dL)	166 ± 89	151 ± 82	.01
CRP (mg/L)	43.63 ± 52.97	26.9 ± 42	<.0001
WBC count	11123 ± 10726	9856 ± 5180	.05
Creat clear (mL/min)	72 ± 34	78 ± 32	.01
Ht (%)	41.4 ± 5.4	41.4 ± 5.0	.93
LDL (mg/dL)	125.36 ± 46.33	127.23 ± 44.6	.59
Prior statin therapy (% hyperlipidemics)	44	42	.21
Current smoking %	49	47	.49
Physical activity (%)	58	66	.30
Hypertension (%)	59	62	.36
Receiving medication for hypertension (%)	41	49	.32
Diabetes mellitus (%)	36	30	.13
Receiving oral medication for diabetes mellitus (%)	31	36	.08
Insulin therapy (%)	30	33	.18

LVSD indicates left ventricular systolic dysfunction; BMI indicates body mass index; Ht indicates hematocrit; Time delay = time in minutes from symptoms onset till hospitalization; Previous CHD: previous known coronary heart disease.

**Table 2:** Results from ANOVA that presents the distribution of clinical and biochemical characteristics among WBC tertile.

	1st tertile	2nd tertile	3rd tertile	*P*
	WBC < 8180/*μ*L	< 8180WBC < 11000/*μ*L	WBC > 11000/*μ*L	

*N*	225	235	237	
Age (yrs)	67.9 ± 11.8	64.2 ± 12.4	61.6 ± 12.5	<.0001
Male sex %	76	80	82	.287
Body mass index (Kg/m^2^)	27.3 ± 4.3	28.4 ± 5.5	27.8 ± 3.9	.132
Time delay (min)	830.8 ± 3421	343.5 ± 763.3	655.7 ± 2178	.251
Presence of MI, %	63	77	90	<.0001
History of CHD, %	45	35	24	<.0001
Troponin I at entry (ng/mL)	5.56 ± 22.68	8.90 ± 25.46	14.87 ± 38.04	.004
Smoking, %	34	54	56	<.0001
Hypertension, %	63	63	58	.480
Diabetes %	35	28	33	.303
Hypercholesterolemia, %	55	60	58	.754
Creatinine clearance	68.33 ± 30.5	79.75 ± 33.2	78.28 ± 33.4	<.0001
Systolic blood pressure at entry (mm Hg)	129.5 ± 26	131 ± 26	133 ± 31	.26
Diastolic blood pressure at entry (mm Hg)	76 ± 12	77 ± 13	77 ± 15	.55
Heart rate at entry (beats/min)	78 ± 15	75 ± 13	90 ± 16	.05

Time delay: time in minutes from symptoms onset till hospitalization; MI: myocardial infarction; History CHD: previous known coronary heart disease.

**Table 3:** Results from ANOVA that presents the distribution of clinical and biochemical characteristics among CRP tertile.

	1st tertile	2nd tertile	3rd tertile	*P*
	CRP < 6.52 mg/L	< 6.52CRP < 28 mg/L	CRP > 28 mg/L	

*N*	207	209	204	
Age (yrs)	63 ± 12.6	63.2 ± 13.4	66.3 ± 12.5	.014
Male sex %	80	77	81	.549
Body mass index (Kg/m^2^)	28.1 ± 5.8	27.7 ± 4.3	27.7 ± 4	.709
Time delay (min)	836.7 ± 3860	609.5 ± 2191	551.7 ± 1600	.721
Presence of MI, %	75	85	90	<.0001
History of CHD, %	31	28	26	.625
Troponin I at entry (ng/mL)	6.08 ± 25.95	11.72 ± 28.05	17.43 ± 45.84	.006
Smoking, %	45	53	51	.221
Hypertension, %	63	56	59	.310
Diabetes %	27	30	35	.180
Hypercholesterolemia, %	62	59	51	.299
Creatinine clearance	79.35 ± 33.7	77.02 ± 33.5	68.96 ± 31.9	.007
Systolic blood pressure at entry (mm Hg)	131 ± 25	131 ± 28	133 ± 32	.97
Diastolic blood pressure at entry (mm Hg)	77 ± 12	76 ± 13	76 ± 15	.68
Heart rate at entry (beats/min)	73 ± 25	80 ± 15	85 ± 16	.28

Time delay: time in minutes from symptoms onset till hospitalization; MI: myocardial infarction; History CHD: previous known coronary heart disease.

**Table 4:** Results from logistic regression model that evaluated the role of CRP and WBC (independent covariates) on the occurrence of LVSD in patients who had had an acute coronary event, after adjustment for several common confounders*.

	Odds ratio	95% confidence interval
Model 1: CRP (per mg/L)	1.06	1.02–1.10
Model 2: WBC (per 1000 count)	1.07	1.02–1.12

*Covariates also used in both models were age, sex, diagnosis of myocardial infarction, creatinine and troponin I levels at entry, systolic, and diastolic blood pressure levels at entry.
